# One health survey of *Enterocytozoon bieneusi* in rural Adana (Türkiye) reveals zoonotic genotypes and two novel ITS genotypes in livestock

**DOI:** 10.1007/s00436-026-08662-w

**Published:** 2026-03-28

**Authors:** Eylem Akdur-Öztürk, Yaseen Majid Salman Al-Adilee, Çağıl Coşkun, Alejandro Dashti, Sergio Sánchez, Eleni Gentekaki, Anastasios D. Tsaousis, Funda Dogruman-Al, David Carmena

**Affiliations:** 1 Department of Medical Parasitology, Faculty of Medicine, Çukurova University, Adana, Türkiye; 2 School of Natural Sciences, University of Kent, Canterbury, Kent, UK; 3 Department of Medical Microbiology, College of Medicine, Ninevah University, Mosul, Ninevah, Iraq; 4 Department of Biophysics, Faculty of Medicine, Çukurova University, Adana, Türkiye; 5 Parasitology Reference and Research Laboratory, Spanish National Centre for Microbiology, Health Institute Carlos III, Majadahonda, Spain; 6 Department of Veterinary Medicine, University of Nicosia School of Veterinary Medicine, Nicosia, Cyprus; 7 Division of Medical Parasitology, Department of Medical Microbiology, Faculty of Medicine, Gazi University, Ankara, Türkiye; 8 Center for Biomedical Research Network (CIBER) in Infectious Diseases, Health Institute Carlos III, Madrid, Spain

**Keywords:** Genotyping, ITS, human, livestock, cattle, sheep, goat, zoonoses, One Health

## Abstract

*Enterocytozoon bieneusi* is the most frequently detected microsporidian in humans and infects a wide range of animals. Data integrating human, animal, and environmental compartments under a One Health framework remains scarce. We conducted a molecular survey in a rural area of Adana Province, south-central Türkiye (October–November 2023). A total of 394 samples were screened, including stool from apparently healthy individuals (*n =* 124) from 64 households and livestock such as cattle (*n =* 75), sheep (*n =* 95), goats (*n =* 60) and environmental samples (lake water, *n =* 24; mud, *n =* 16). Detection and genotyping targeted the Internal Transcribed Spacer (ITS) region by nested PCR and Sanger sequencing. No human or environmental samples were positive, this result may be influenced by sampling limitations, seasonality, and methodological sensitivity rather than reflecting true absence. Overall *E. bieneusi* prevalence in livestock was 5.2% (12/230; 95% CI, 2.6–8.6). By host, prevalence was 8.4% in sheep (8/95; 95% CI, 3.7–15.9), 3.3% in goats (2/60; 95% CI, 0.4–11.5), and 2.7% in cattle (2/75; 95% CI, 0.3–9.3); differences were not statistically significant. Sequence analysis identified three known genotypes: BEB6 (6/12), J (1/12), and Type IV (1/12), and two novel genotypes (ShTrEb1, ShTrEb2; 2/12 each). J and Type IV were restricted to cattle; BEB6 and ShTrEb1 occurred in sheep and goats; ShTrEb2 was detected only in sheep. Phylogenetic analysis placed Type IV in Group 1 and BEB6, J, ShTrEb1, and ShTrEb2 in Group 2. Although humans and environmental matrices tested negative in this study, the detection of genotypes in livestock should be interpreted carefully and highlights the need for further studies to clarify potential transmission pathways. Larger, seasonally stratified surveys with standardized environmental methods are warranted to better understand regional transmission dynamics.

**Supplementary Information:**

The online version contains supplementary material available at 10.1007/s00436-026-08662-w.

## Introduction

Microsporidia belong to a phylum of obligate intracellular, spore-forming microeukaryotes comprising nearly 1,700 species that infect both vertebrate and invertebrate hosts, 17 of which have been reported in humans (Han et al. 2021). Among them, *Enterocytozoon bieneusi* is the most prevalent species, accounting for approximately 90% of global human intestinal microsporidiosis (Matos et al. 2012). In immunocompromised patients, such as organ transplant recipients or the elderly, *E. bieneusi* typically causes long-term diarrhoea and weight loss (Matos et al. 2012; Han et al. 2021). In contrast, infections in immunocompetent individuals are often asymptomatic, although they may occasionally present with self-limiting diarrhoea or impaired nutrient absorption (López-Vélez et al. 1999; Sak et al. 2011a, b). Transmission occurs via the faecal-oral route by ingestion of environmentally resistant spores that can persist under adverse conditions. This resilience facilitates the occurrence of waterborne or foodborne outbreaks of microsporidiosis (Decraene et al. 2012; Li and Xiao 2020; Michlmayr et al. 2020; Bourli et al. 2023).

Analysis of the internal transcribed spacer (ITS) region of the ribosomal RNA gene has revealed more than 500 distinct genotypes of *E. bieneusi*, which are classified into 15 phylogenetic groups. Of these, Groups 1 and 2 include genotypes with zoonotic potential, sporadic human infections caused by genotypes outside these groups have been reported, particularly in immunocompromised individuals (Wang et al. 2013; Li et al. 2018; Jiang et al. 2024). *Enterocytozoon bieneusi* is widely distributed among wild, farmed, and domestic animals worldwide, raising the question about the extent to which these species serve as natural reservoirs of the parasite (Santín and Fayer 2009; Li et al. 2018). Therefore, studies that simultaneously assess human, animal, and environmental samples within a One Health perspective are becoming increasingly important for comprehensively mapping the host range, transmission routes, and geographical distribution of *E. bieneusi.*

The epidemiology of *E. bieneusi* in Türkiye has only recently begun to be investigated (Table 1) (Ercan et al. 2025). In humans, *E. bieneusi* infections have been reported in cancer patients undergoing chemotherapy, bone marrow transplant recipients, and in both diarrheic and non-diarrheic patients regardless of immune status. Reported prevalence rates ranged from 2 to 17% when detected by immunofluorescence assays to 8–52% when PCR-based methods were used (Çetinkaya et al. 2015; Hamamcı et al. 2015; Oğuz Kaya et al. 2018; Aydemir et al. 2023; Aksoy Gökmen et al. 2024). Using the latter assay, detection of *E. bieneusi* spore DNA in livestock species including camels, cattle, horses, sheep, and water buffaloes at prevalence rates of 3–19% (Bilgin et al. 2020; Yıldırım et al. 2020; Onder et al. 2022; Apaydın et al. 2023; Şimşek et al. 2025; Öncü Öner et al. 2025), in avian species including chickens, budgerigars, and pigeons at prevalence rates of 4–14% (Ercan et al. 2020; Pekmezci et al. 2020, 2021), and stray cats at prevalence rates of 6–50% (Pekmezci et al. 2019; Erkun Alak et al. 2023; Sürgeç et al. 2023). Domestic flies have also been demonstrated to act as mechanical carriers of spores of *E. bieneusi* (Ercan et al. 2024). Regarding environmental samples, *E. bieneusi* has been detected in surface waters and paddock water containers used for watering livestock (Öncü Öner et al. 2025). Information on the genotypes of *E. bieneusi* circulating in humans and animals is even scarcer, particularly for the former. In humans, genotypes D and Type IV have been identified in a limited number of clinical samples (Aksoy Gökmen et al. 2024), whereas a much wider diversity has been observed in animal species (Table 1).

**Table 1:** Prevalence and genotype distribution of *Enterocytozoon bieneusi* in human, animal, and environmental samples in Türkiye, 2015–2025

Source	Population/type of sample	Area	Detection method	Samples (*n*)	Infection rate (%)	Genotypes (*n*)	Reference	
Human	BMTR, diarrhoea	Kayseri	IFA	147	4.8	ND	Çetinkaya et al. (2015)	
	BMTR, no diarrhoea			53	5.7	ND		
	Healthy controls			80	2.5	ND		
	Oncologic, diarrhoea	Kayseri	IFA	51	11.8	ND	Hamamcı et al. (2015)	
	Oncologic, no diarrhoea			42	16.6	ND		
	Healthy controls			30	3.3	ND		
	Diarrheic patients	Ankara	Chem, nPCR	200	3.5	ND	Oğuz Kaya et al. (2018)	
	Immunosuppressed, diarrhoea	Various	IFA, RT-PCR	88	52.2	ND	Aydemir et al. (2024)	
	Immunocompetent, diarrhoea			38	26.1	ND		
	Immunosuppressed, no diarrhoea			38	21.7	ND		
	Immunocompetent, no diarrhoea			36	0.0	ND		
	Oncologic, diarrhoea	İzmir	RT-PCR, nPCR	94	25.5	D (1), Type IV (2)	Aksoy Gökmen et al. (2024)	
	Immunocompetent, diarrhoea			50	8.0	Type IV (1)		
	Immunocompetent, no diarrhoea			50	10.0	Type IV (1)		
Animal	Camel	Various	nPCR	110	2.7	CamelEb (3)	Şimşek et al. (2025)	
	Cattle	Sivas	nPCR	150	19.3	ERUSS1 (24), ERUSS2 (1), ERUSS3 (1), ERUSS4 (1), N (2)		Bilgin et al. (2020)
	Cattle		nPCR	47	12.8	NG1-2 (2), NG5-7 (3), Type IV (1)	Öncü Öner et al. (2025)	
	Horses	Kayseri	nPCR	300	18.7	BEB6 (8), ERUH2 (6), ERUH3 (5), ERUH4 (4), ERUH5 (6), ERUH6 (3), ERUH7 (2), ERUSS1 (24)	Yıldırım et al. (2020)	
	Sheep	Van	nPCR	200	8.0	BEB6 (16)	Apaydın et al. (2023)	
	Water buffaloes	Various	nPCR	300	2.6	J (3), YNDCEB-90 (5)	Onder et al. (2022)	
	Chicken	Various	nPCR	300	7.3	ERUNT1 (21), ERUSS1 (1)	Ercan et al. (2020)	
	Cat	Samsun	nPCR	72	5.5	D (2), Type IV (2)	Pekmezci et al. (2019)	
	Cat	İzmir	RT-PCR,	339	50.2	ND	Erkun Alak et al., (2023)	
	Cat	İzmir	nPCR	170^a^	–	D (3), Type IV (44)	Sürgeç et al. (2023)	
	Budgerigars	Samsun	nPCR	143	3.5	N (2), TURKM1 (3)	Pekmezci et al. (2020)	
	Pigeons	Samsun	nPCR	250	14.0	Peru 6 (35)	Pekmezci et al. (2021)	
	Domestic flies	Several	nPCR	850	2.4	AEUEb (3), BEB6 (4), BEB8 (6), Type IV(1)	Ercan et al. (2024)	
Environmental	Water	Manisa	nPCR	41	22.0	NG3-4 (2), NG8-11 (5), Type IV (2)	Öncü Öner et al. (2025)	

^a^Samples positive for *E. bieneusi* from a previous study

*BMTR* Bone marrow transplant recipient, *Chem* Chemiluminescence, *IFA* Immunofluorescence assay, *ND* Not determined, *nPCR* Nested PCR, *RT-PCR* Real-Time PCR

Considering the scarcity of field-based One Health surveys on *E. bieneusi*, this local baseline study aimed to investigate the presence and genetic diversity of the pathogen simultaneously in apparently healthy individuals, livestock, and environmental samples in a rural area of Adana Province, south-central Türkiye, and to investigate the transmission dynamics using a One Health approach.

## Materials and methods

### Study design and sampling area

**Fig. 1:**
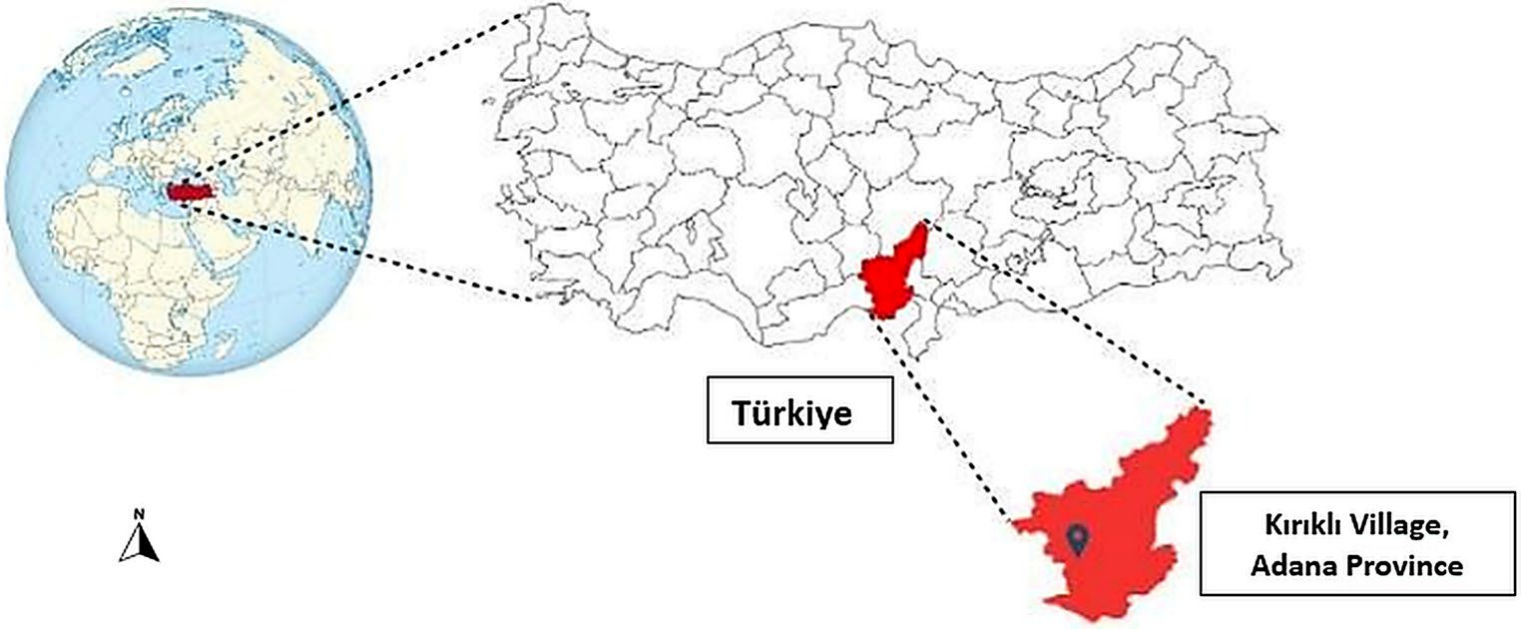
Map showing the location of Karaisali district, Adana Province, Türkiye. The sampling area is indicated with a pin

This observational field study was conducted in Kırıklı (37°10′N, 35°14′E), a rural village with a population of 582 located in the Karaisalı district of Adana Province, Türkiye (Fig. 1). The local economy relies primarily on agriculture and animal husbandry. The area has a Mediterranean climate characterized by extremely hot, humid summers and mild, wet winters. The study site includes the shores of the Seyhan Dam Lake, where seasonal fluctuations in water level create areas intensively used by both humans (e.g., picnics, camping) and animals (e.g., grazing). In this survey, we used DNA samples of human, animal, and environmental origin from a previous study conducted by our research group, which investigated the transmission dynamics of *Blastocystis* within a One Health framework (Öztürk et al. 2025).

### Sample collection and processing

A purposive sampling strategy was used to identify eligible households (Işıklar 2019), after which individual stool samples were randomly collected from 124 apparently healthy residents across 64 households from a defined population of 540 residents in the study area. The required sample size was calculated using a single-proportion formula with finite population correction at a 95% confidence level (*p* = 0.5). Of these, 90 participants belonged to households (*n* = 46) actively engaged in animal husbandry, while 34 participants came from households (*n* = 18) without animals and reported no animal ownership for at least six months prior to sampling. None of the participants reported diarrhoea or bloody stools at sampling. Each volunteer who agreed to participate in the survey was provided with a labelled, sterile faecal collection container and given instructions on how to safely collect the sample. For participants of paediatric age, sample collection was carried out by parents or legal guardians. The first stool sample of the day was delivered to the health centre by volunteers and systematically retrieved by the research team during official working hours. Only basic sociodemographic variables (sex, age, sampling location) were available for this study.

Individual faecal samples from livestock species, including cattle (*n* = 75), sheep (*n* = 95), and goats (*n* = 60), were randomly collected in 46 households, with all animal sampling procedures carried out by members of the research team. To minimize environmental contamination, samples were collected immediately after animals’ defecation and transferred into pre-labelled faecal collection containers. There was no macroscopic blood or diarrhoea in any of the selected animal faecal samples.

Twenty-four dam lake water samples with varying turbidities and 16 mud samples were collected from sites around the Seyhan Dam Lake. This site is subject to intensive human activity, including recreational uses such as camping, fishing, and picnicking, as well as animal grazing and irrigation for livestock and agricultural purposes. Water samples were taken from the lake, its tributaries, and standing water using sterile 1-L containers. Samples were left to settle overnight at room temperature (Jinatham et al. 2021). The supernatant was drained until ~ 50 mL of sediment remained, which was transferred to 15-mL tubes and centrifuged at 500 g for 10 min. The supernatant was then discarded, and the pellet kept. Mud samples (~ 15 g) were collected from the same sites.

Samples were collected between October and November 2023 and transported in refrigerated boxes to the Medical Parasitology Laboratory of the Faculty of Medicine (Çukurova University). Human and animal faecal samples and environmental (water and mud) samples were preserved in DNA/RNA Shield™ (Zymo Research, Freiburg, Germany) at a 1:2 ratio and stored at 4 °C until further processing. Stool and mud samples were preserved directly at this ratio after collection, while water samples were first pelleted (see above) and the resulting pellet stored at a 1:2 ratio. DNA extraction (see below) was conducted within 1–3 months after sample collection.

The One Health sampling campaign (humans, livestock and environmental matrices) and sample preservation/DNA extraction were performed as part of our previously published rural Türkiye survey investigating Blastocystis transmission dynamics (Öztürk et al. 2025). Briefly, samples were collected in October–November 2023 from the same study site and households/animals and stored in DNA/RNA Shield prior to genomic DNA extraction using the PureLink Microbiome DNA Purification Kit, as described in that study. The present manuscript represents a secondary analysis of these archived DNA extracts (no additional field sampling was undertaken), in which we screened for and genotyped *Enterocytozoon bieneusi* using nested PCR of the ITS region followed by Sanger sequencing and phylogenetic analysis.

### Genomic DNA extraction and purification

The genomic DNA was extracted from 200 µL of thoroughly vortexed (human, animal or environmental) samples in DNA/RNA Shield™ (Zymo Research) using the PureLink™ Microbiome DNA Purification Kit (Thermo Fisher Scientific, Carlsbad, CA, USA) following the manufacturer’s protocol. The purified DNA was eluted in 100 µL of elution buffer and stored at − 20 °C until further molecular testing.

### Molecular detection and characterization of *Enterocytozoon bieneusi*

Detection of *E. bieneusi* DNA was accomplished using a nested PCR targeting the internal transcribed spacer (ITS) region and the flanking large and small subunit of the ribosomal RNA gene (~ 390 bp). The selected fragment length follows the globally accepted nomenclature system used in molecular epidemiological studies of this species (Buckholt et al. 2002; Santín and Fayer 2009; Jiang et al. 2024).

The first-round PCR was performed with the primer pair EBITS3 (5´–GGTCATAGGGATGAAGAG–3´) and EBITS4 (5´–TTCGAGTTCTTTCGCGCTC–3´), while the second-round PCR was performed using the primer pair EBITS1 (5´–GCTCTGAATATCTATGGCT–3´) and EBITS2.4 (5´–ATCGCCGACGGATCCAAGTG–3´) to produce 390-bp PCR amplicon. Both first- and second-round PCR reactions were performed in a final volume of 20 µL, including 1 µL of template DNA, 1 µL of each 10 µM primer, 4 µL of FirePol Master Mix (Solis BioDyne, Tartu, Estonia), and 13 µL of nuclease-free water. A negative control containing nuclease-free water and a positive control containing a laboratory-confirmed *E. bieneusi* genomic DNA were used in each run. PCR reactions were conducted on a MiniAmp Plus thermal cycler (Applied Biosystems Foster City, CA, USA). Cycling conditions for the first- and second-round PCRs were as follows: an initial denaturation at 95 °C for 4 min, followed by 35 cycles of denaturation at 95 °C for 30 s, annealing at 53 °C for the first-round PCR and 55 °C for the second-round PCR for 45 s, and extension at 72 °C for 1 min. A final extension step was performed at 72 °C for 7 min. Second-round PCR products were analysed by electrophoresis on a 1.2% agarose gel stained with SYBR safe DNA gel stain (Invitrogen, Waltham, MA, USA) and visualized under a UV transilluminator.

### Sequencing analysis

DNA sequencing was carried out using the internal EBITS1/EBITS2.4 primer set on all samples that yielded amplicons of the expected size in the second-round PCR. Reactions were carried out using the BigDye^®^Terminator chemistry on an ABI PRISM 3500 automated DNA sequencer (Applied Biosystems Foster City, California). Raw sequences were examined with the Chromas Lite version 2.1 software (http://chromaslite.software.informer.com/2.1) to generate consensus sequences. These sequences were compared with reference sequences deposited at the National Center for Biotechnology Information using the BLAST tool (http://blast.ncbi.nlm.nih.gov/Blast.cgi). *Enterocytozoon bieneusi* genotypes were assigned according to the established nomenclature system based on ITS sequence data (Santín and Fayer 2009). Sequences generated in the present study were deposited in the GenBank public repository database under accession numbers PX442339–PX442345.

### Phylogenetic analysis

The evolutionary history of the ITS sequences generated in this study was inferred using the Maximum Likelihood (ML) method under the General Time Reversible model (Nei et al. 2000). For comparative purposes, sequences representing human, animal, and/or environmental genotypes spanning all 15 phylogenetic Groups of *E. bieneusi* were included in the analysis. Initial trees for the heuristic searches were obtained by applying the Neighbor-Joining method to a matrix of pairwise distances estimated with the Maximum Composite Likelihood (MCL) approach. A discrete Gamma distribution was used to model evolutionary rate differences among sites. All positions containing gaps and missing data were eliminated. Evolutionary analyses were conducted in MEGA X (Kumar et al. 2018).

### Statistical analysis

Categorical variables were compared using the Fisher-Freeman-Halton exact test, as more than 50% of the contingency table cells had expected counts less than 5. Statistical significance was set at *p* < 0.05.

## Results

### Demographic features of the human participants

Among the 124 participants, 58 (46.8%) were female and 66 (53.2%) were male. The mean age was 44.7 ± 18.2 years (median: 48; range: 6–82).

### Prevalence of *Enterocytozoon bieneusi*

In this study, a sample was considered *E. bieneusi*-positive only if confirmed by Sanger sequencing. Based on this criterion, none of the 124 human stool samples or 40 environmental (water and mud) samples yielded amplicons of the expected size by nested PCR and were regarded as negative. Twenty livestock faecal samples yielded amplicons compatible with *E. bieneusi*. Eight of them corresponded to faint bands on gel that generated unreadable Sanger sequencing data and were regarded as negative. Therefore, the overall prevalence of *E. bieneusi* in livestock was estimated at 5.2% (12/230; 95% CI: 2.6–8.6). By species, *E. bieneusi* infections were more prevalently found in sheep (8.4%, 8/95; 95% CI: 3.7–15.96), followed by goats (3.3%, 2/60; 95% CI: 0.4–11.5), and cattle (2.7%, 2/75; 95% CI: 0.3–9.3). Differences between host species were not statistically significant (Fisher-Freeman-Halton exact test, *P* = 0.198).

### Genotyping of *Enterocytozoon bieneusi*

Sequence analyses of the 12 livestock isolates confirmed as *E. bieneusi*-positive revealed the presence of three known genotypes including BEB6 (50.0%, 6/12), J, and Type IV (8.3%, 1/12 each). Two novel *E. bieneusi* genotypes were found and named ShTrEb1 and ShTrEb2 (16.7%, 2/12 each). Genotype ShTrEb1 varied from CHG3 by a single nucleotide polymorphism (SNP) at the ITS region (T30C) when aligned with reference sequence KP262362. Genotype ShTrEb2 varied from BEB6 by a single SNP at the ITS region (C153T) when aligned with reference sequence EU153584. Genotypes J and Type IV were identified only in cattle, EB6 and ShTrEb1 were each found in one goat, whereas ShTrEb2 was detected only in two sheep (Table 2). The identification of the novel ShTrEb1 and ShTrEb2 sequences in two different animals, each in independent PCR reactions, provides additional support for the robustness of our sequencing data. No ambiguous (double peak) positions were found at chromatogram inspection, indicating absence of mixed infections by two or more genotypes of *E. bieneusi*. According to farm of origin, BEB6 was detected in a goat and two sheep from the same farm (R27), as well as in two sheep from a different farm (R25). The remaining seven *E. bieneusi* isolates were identified in single animals from independent farms, with no evidence of cross-species transmission. Genotype distributions at the livestock and household levels are summarized in Supplementary Table S1.

**Table 2:** Molecular frequency and diversity of *Enterocytozoon bieneusi* in livestock species in the present study

Host species	Genotype	Isolates (*n*)	Reference sequence	Single nucleotide polymorphisms	GenBank accession number
Cattle	J	1	AF135837	None	PX442339
	Type IV	1	AF242478	None	PX442340
Goats	BEB6	1	EU153584	None	PX442341
	ShTrEb1	1	–	–	PX442342
Sheep	BEB6	5	EU153584	None	PX442343
	ShTrEb1	1	–	–	PX442344
	ShTrEb2	2	–	–	PX442345

**Fig. 2:**
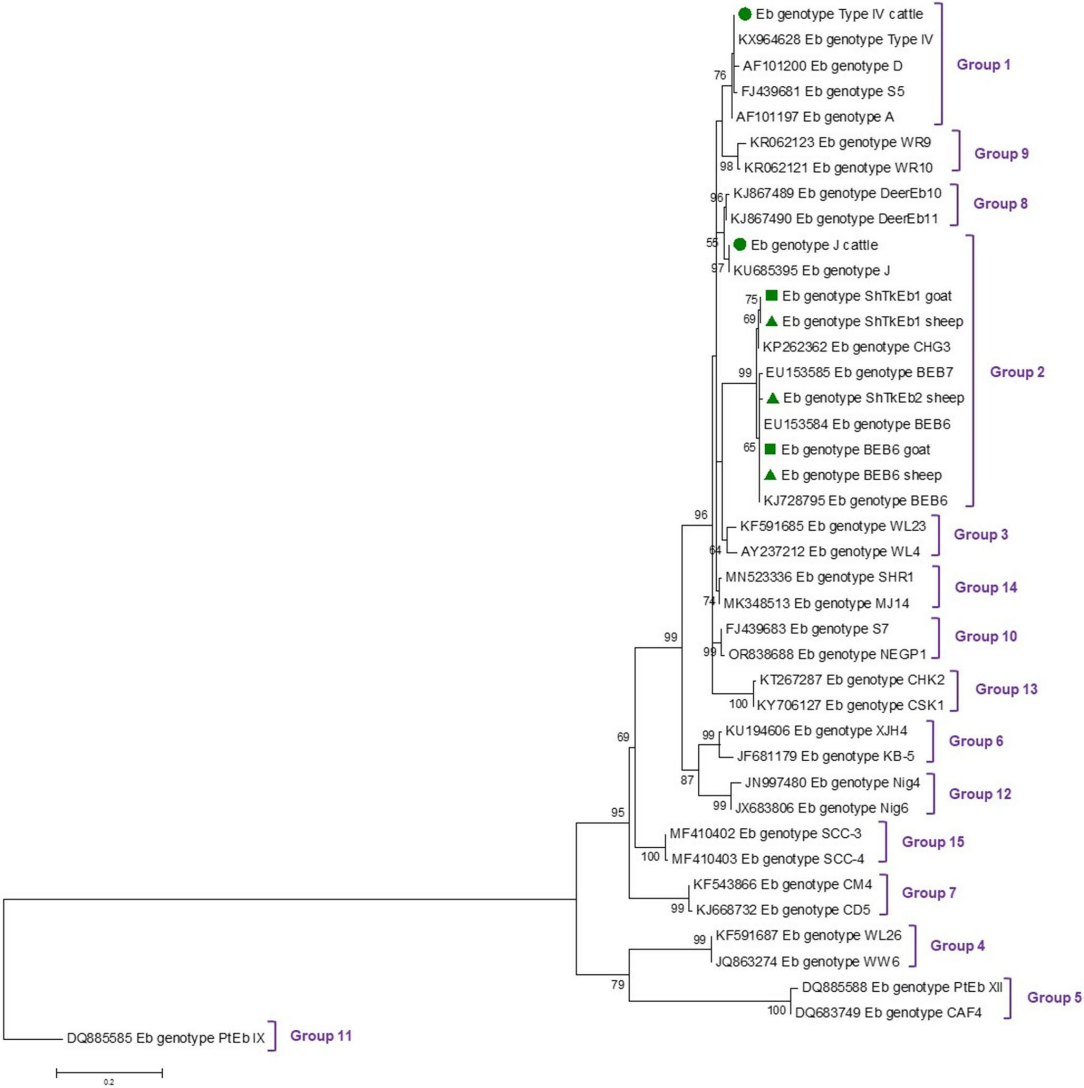
Phylogenetic relationships among *E. bieneusi* genotypes identified in this study using the complete ITS sequence (243 bp) and a selection of genotypes identified in humans and/or animals to cover all 15 Groups of *E. bieneusi*. Sequences generated in this study are represented with green filled circles (cattle), squares (goats) or triangles (sheep). Numbers at the nodes represent the bootstrap values with more than 50% bootstrap support from 1000 replicates

Our phylogenetic analysis showed that all four *E. bieneusi* genotypes found here cluster together in well-defined Groups: Type IV within Group 1, and BEB6, J, ShTrEb1 and ShTrEb2 within Group 2 (Fig. 2).

## Discussion

This study investigated the molecular epidemiology of *E. bieneusi* in a rural community of Adana province (south-central Türkiye) where such data were previously unavailable. The main strengths of the study were: (i) the adoption of a One Health framework that considers the interactions among villagers, livestock, and shared water resources—an approach not previously applied in Türkiye, (ii) the inclusion of asymptomatic and immunocompetent individuals—an underrepresented Group in most microsporidial studies, (iii) the expansion of current knowledge on suitable host species in Türkiye.

A recent meta-analysis reported a global positivity rate of 6.6% for *E. bieneusi* in humans (Wang et al. 2024). Prevalence was significantly higher among HIV-infected individuals (11.5%) and patients with diarrhoea (16.5%) compared to asymptomatic individuals (6.5%). These findings indicate that host immune status and clinical symptoms are key factors influencing infection risk and detection. Most human infections by *E. bieneusi* are caused by genotypes belonging to zoonotic Groups 1 (82.1%) and 2 (7.9%), with the former showing the broadest host range (Wang et al. 2024). However, available data indicate host adaptation in most genotypes in Groups 3 to 15 and three outlier genotypes (CSK2, Nig5, SW3) (Jiang et al. 2024), have also been less frequently reported in both humans and animals, highlighting the zoonotic potential of these genotypes and the need to expand human–animal studies (Li et al. 2018; Jiang et al. 2024; Wang et al. 2024).

In Türkiye, only five studies have investigated the presence of *E. bieneusi* in humans, reporting an overall prevalence of 11.2% (103/917) (Table 1). All of them targeted vulnerable clinical populations, primarily immunocompromised individuals such as cancer patients undergoing chemotherapy, bone marrow transplant recipients, and patients with malignant solid tumours. To date, no information exists on the epidemiology of this pathogen in the general population. In the present study, none of the 124 apparently healthy individuals tested positive for *E. bieneusi*, suggesting that, if present, the parasite circulates at very low prevalence and/or at loads below the PCR detection threshold (Ghosh and Weiss 2009).

In the present study, none of the 124 apparently healthy individuals tested positive for *E. bieneusi*. Given the cross-sectional design and the availability of a single stool specimen per participant, this finding should be interpreted conservatively as an absence of sequence-confirmed positives at the time of sampling rather than evidence of absence in the community. Intermittent spore shedding and temporal variation in parasite burden can reduce the probability of detection when only one sample is analysed per individual. Importantly, the nested ITS PCR performed as expected, with appropriate controls included in each run and successful amplification in livestock samples, supporting the validity of the assay. With 0/124 detections, the 95% upper bound on point prevalence is ~ 2.4% (rule-of-three), which remains compatible with low-level carriage in the general population. By contrast, subclinical infections have been reported elsewhere, with prevalence rates ranging from 1 to 9% in apparently healthy individuals in the Czech Republic (Sak et al. 2011a, b) and in asymptomatic children in Mozambique (Muadica et al. 2020) and Zambia (Mutengo et al. 2025). Additionally, the failure to detect the parasite may be related to intermittent spore shedding and the analysis of only a single stool sample per individual, which could reduce the likelihood of parasite detection. Furthermore, while nested PCR targeting the ITS region has been widely used with good sensitivity in previous studies, potential factors such as poor DNA quality or the presence of non-specific amplification products can also lead to undetection.

Global meta-analyses have estimated the overall prevalence of *E. bieneusi* at 14.0% (95% CI: 11.5–17.0) in cattle, 17.4% (95% CI: 11.8–25.0) in sheep, and 16.3% (95% CI: 11.3–22.8) in goats (Taghipour et al. 2021, 2022). These studies also showed that genotypes BEB4, J, and I were most identified in cattle, while BEB6 and COS-1 predominated in sheep, and CHG3 and BEB6 were more frequent in goats. In the present study, the overall prevalence of *E. bieneusi* in the surveyed livestock population was 5.2%, including 8.4% in sheep, 3.3% in goats, and 2.7% in cattle. Previous molecular-based epidemiological surveys conducted in Türkiye reported prevalence rates of *E. bieneusi* ranging from 8.0% in sheep (Apaydın et al. 2023) to 12.8–19.3% in cattle (Bilgin et al. 2020; Öncü Öner et al. 2025), but no data from goats were previously available in the country. Our molecular data also showed interesting results: The most noteworthy finding of this study is the identification of two novel *E. bieneusi* genotypes, named ShTrEb1 and ShTrEb2 in ovines and caprines. While genotypes BEB6 and CHG3 were detected in ovines and caprines, genotypes J and Type IV were identified only in cattle. The predominance of BEB6 in sheep and goats, and (to a lower extent) of J in cattle, aligns with global trends reported in meta-analysis studies (Taghipour et al. 2021, 2022), suggesting that certain genotypes exhibit a preference for specific herbivore hosts. Additionally, all four genotypes found here belong to zoonotic Groups 1 and 2. Of note, other genotypes previously described in Turkish livestock such as ERUH2-7, ERUSS1-4, N, TURKM1, and YNDCEB-90 (see Table 1) were not detected in the livestock population surveyed in the present survey. Taken together, these findings expand current knowledge on the epidemiology of *E. bieneusi* in Türkiye and offer insights into cross-species transmission among farm animals and, potentially, humans.

Environmental sources such as surface water, soil, and fresh produce are thought to play an important role in the transmission of *E. bieneusi* and serve as reservoirs for human and animal infections (Li and Xiao 2020). Indeed, outbreaks of human microsporidiosis by *E. bieneusi* of waterborne (Hunter 2000) and foodborne (Decraene et al. 2012; Michlmayr et al. 2020) origin have been documented in countries such as France, Sweden and Denmark. None of the water and mud samples analysed in the present study tested positive for *E. bieneusi*. In this regard, it should be noted that no standardized method currently exists for the detection and quantification of microsporidial spores in environmental matrices. In practical terms, this means that the procedures presently available are hindered by low detection limits and variable reproducibility and repeatability rates, all of which affect their overall diagnostic performance. In the absence of standardized protocols, we adopted a cost-effective and practical approach based on the combined use of water and mud sediments with PCR, an approach successfully used by our team for the detection of enteric microeukaryotes in other settings (Jinatham et al. 2021). Of note, DNA of *E. bieneusi* was detected in 22.0% (9/41) of environmental (drinking water containers, feeder surfaces, milk warming tanks, and towels) samples in a dairy farm study recently conducted in Türkiye, although the survey did not provide detailed information on the detection methodology (Öncü Öner et al. 2025). The high positivity observed in that farm environment was attributed to the elevated pathogen load resulting from the constant and close contact between animals, feed, and water sources, which together contributed to the transmission and maintenance of the pathogen. In contrast, the samples in our study were collected from open areas, where environmental factors such as rainfall, wind, and natural water flow may reduce spore density through dilution and dispersal. These variables, together with the technical limitations described above, could therefore explain (at least practically) the absence of *E. bieneusi* in the environmental samples analysed in this study. Multicenter longitudinal studies with standardized methods and study designs are needed to demonstrate the impact of geographic differences on the prevalence and genotype distribution of *E. bieneusi.*

Environmental non-detection likely reflects sensitivity limits rather than absence. In open matrices, spore concentrations are expected to be low and spatially heterogeneous; the effective volume processed, matrix turbidity, and polymerase inhibitors can further reduce detection probability even with mechanical lysis and inhibitor-removal steps. Additionally, the absence of a filtration step prior to DNA extraction may have influenced the sensitivity of detection, Our conservative rule, counting only sequence-confirmed amplicons as positive, adds specificity at the cost of sensitivity. These factors together provide a parsimonious explanation for the observed ‘animal-positive/environment-negative’ pattern at a single time point.

From a One Health perspective, the detection of *E. bieneusi* in cattle, sheep, and goats (but not in humans or environmental samples) raises epidemiological concerns. The presence of zoonotic genotypes (BEB6, J, Type IV) suggests livestock may act as sources of environmental contamination, despite the fact this extent could not be confirmed here. Spores shed in the faeces of infected animals could enter surface waters via farm runoff, posing a risk of food contamination through irrigation. However, in the study area, faeces are sun-dried for fertilizer. This practice, combined with extreme regional temperatures, may reduce spore viability and limit water contamination. Taken together, these findings highlight the convenience of early detection and ongoing monitoring of *E. bieneusi* in animals to protect human and environmental health and support further One Health–based field research across diverse regions.

This research has several limitations; (i) the most significant of these is that seasonal factors cannot be assessed because the study had a cross-sectional (transversal) design and was not longitudinal, (ii) the study was conducted in a single regional setting, the findings may not be representative of other geographic regions or epidemiological scenarios in Türkiye, (iii) limiting the sample to farm animals also led to the oversight of potential differences in other animal species, (iv) lack of standardised protocols for the testing of environmental samples very likely influenced negatively their diagnostic performance. Our conservative rule (accepting only sequence-confirmed amplicons) may bias prevalence downward, but it safeguards against false positives and ensures that genotype calls and phylogeny rest on verifiable sequence data.

From a One Health perspective, the compartment pattern is ecologically coherent: enclosed livestock settings concentrate faecal deposition, whereas surrounding soils and waters are dilution-prone sinks. To move beyond plausibility, future work should (i) include temporal replication across dry/wet periods; (ii) process larger volumes with validated spike-and-recovery to quantify method performance per matrix; and (iii) apply qPCR/ddPCR with internal controls to place concentration bounds on non-detections. These steps will allow us to convert qualitative explanations into quantitative estimates of detection probability and transmission risk.

## Conclusions

Although *E. bieneusi* was not detected in human or environmental samples, the present study identified genotypes in farm animals, indicating a limited zoonotic potential and suggesting that these animals may act as reservoirs in the study area. These findings contribute to the limited data on the molecular epidemiology of *E. bieneusi* in Türkiye and underscore the need for future studies across different seasons, broader geographic regions, and extended sampling periods within a One Health framework.

## Supplementary Information

Below is the link to the electronic supplementary material.

Supplementary Material 1

Supplementary Material 2

## Data Availability

Available from the corresponding author on reasonable request.

## References

[CR1] Aksoy Gökmen A, Öncü Öner T, Erkunt Alak S et al (2024) Molecular prevalence and genotypes of Enterocytozoon bieneusi in cancer patients under chemotherapy in Aegean region of Türkiye. BMC Microbiol 24:223. 10.1186/s12866-024-03369-3 38926815 10.1186/s12866-024-03369-3 PMC11202370

[CR2] Apaydın MH, Yetişmiş G, Karabulut F et al (2023) Molecular prevalence and phylogenetic characterization of Enterocytozoon bieneusi in sheep in the Van Region. Turkiye Parazitol Derg 47:64–70. 10.4274/tpd.galenos.2022.76476 37249107 10.4274/tpd.galenos.2022.76476

[CR3] Aydemir S, Halidi AG, Ekici A (2023) Investigation of the presence of Enterocytozoon bieneusi and Encephalitozoon intestinalis in immunosuppressed patients with diarrhoea by IFA and real time PCR methods. Indian J Med Microbiol 44:100362. 10.1016/j.ijmmb.2023.02.005 37356849 10.1016/j.ijmmb.2023.02.005

[CR4] Bilgin T, Usluğ S, Karademir GK et al (2020) Molecular prevalence and phylogenetic characterization of Enterocytozoon bieneusi in healthy cattle. Turkiye Parazitol Derg 44:36–42. 10.4274/tpd.galenos.2020.6851 32212592 10.4274/tpd.galenos.2020.6851

[CR5] BourliP, Eslahi AV, Tzoraki O et al (2023) Waterborne transmission of protozoan parasites: a review of worldwide outbreaks - an update 2017–2022. J Water Health 21:1421–1447. 10.2166/wh.2023.094 37902200 10.2166/wh.2023.094

[CR6] Buckholt MA, Lee JH, Tzipori S (2002) Prevalence of Enterocytozoon bieneusi in swine: an 18-month survey at a slaughterhouse in Massachusetts. Appl Environ Microbiol 68:2595–2599. 11976142 10.1128/AEM.68.5.2595-2599.2002 PMC127518

[CR8] Decraene V, Lebbad M, Botero-Kleiven S et al (2012) First reported foodborne outbreak associated with microsporidia, Sweden, October 2009. Epidemiol Infect 140:519–527. 21733266 10.1017/S095026881100077X PMC3267097

[CR9] Ercan N, Duzlu O, Yildirim A (2020) Molecular detection and genotyping of microsporidia species in chickens in Turkey. Comp Immunol Microbiol Infect Dis 72:101516. 10.1016/j.cimid.2020.101516 32663701 10.1016/j.cimid.2020.101516

[CR11] Ercan N, Yildirim A, Duzlu O et al (2024) Identification and distribution of some medico-veterinary important pathogens in muscid flies in two geographical regions of Türkiye. Med Vet Entomol 38:440–448. 10.1111/mve.12734 38864653 10.1111/mve.12734

[CR10] Ercan N, Yildirim A (2025) Molecular prevalence and genotype distribution of *Enterocytozoon bieneusi* in different hosts in Türkiye: A systematic review and meta-analysis. Acta Vet Eurasia 51. 10.5152/actavet.2025.24054

[CR12] Erkunt Alak S, Can H, Değirmenci Döşkaya A et al (2023) Molecular prevalence of Enterocytozoon bieneusi in stray cats of İzmir, Türkiye. Comp Immunol Microbiol Infect Dis 100:102037. 10.1016/j.cimid.2023.102037 37556942 10.1016/j.cimid.2023.102037

[CR7] Çetinkaya Ü, Hamamcı B, Kaynar L et al (2015) Investigation of the presence of Encephalitozoon intestinalis and Enterocytozoon bieneusi in bone marrow transplant patients by IFA-MAbs method. Mikrobiyol Bul 49:432–438. 10.5578/mb.9809 26313284 10.5578/mb.9809

[CR13] Ghosh K, Weiss LM (2009) Molecular diagnostic tests for microsporidia. Interdiscip Perspect Infect Dis 2009:926521. 10.1155/2009/926521 19657457 10.1155/2009/926521 PMC2719812

[CR14] Hamamcı B, Çetinkaya Ü, Berk V et al (2015) Prevalence of Encephalitozoon intestinalis and Enterocytozoon bieneusi in cancer patients under chemotherapy. Mikrobiyol Bul 49:105–113. 10.5578/mb.8787 25706736 10.5578/mb.8787

[CR15] Han B, Pan G, Weiss LM (2021) Microsporidiosis in Humans. Clin Microbiol Rev 34:e0001020. 10.1128/CMR.00010-20 34190570 10.1128/CMR.00010-20 PMC8404701

[CR16] HunterPR (2000) Waterborne outbreak of microsporidiosis. J Infect Dis 182:380–381 10.1086/315654 10.1086/315654 10882635 10.1086/315654

[CR17] Işıklar E (2019) In: Özmen A, Şeniş BF (eds) İstatistik. Anadolu Üniversitesi, Eskişehir, pp 155–176

[CR18] Jiang S, Yu S, Feng Y (2024) Widespread distribution of human-infective Enterocytozoon bieneusi genotypes in small rodents in northeast China and phylogeny and zoonotic implications revisited. Acta Trop 253:107160. 10.1016/j.actatropica.2024.107160 38408590 10.1016/j.actatropica.2024.107160

[CR19] Jinatham V, Maxamhud S, Popluechai S (2021) Blastocystis One Health approach in a rural community of northern Thailand: Prevalence, subtypes and novel transmission routes. Front Microbiol 12:746340. 10.3389/fmicb.2021.746340 34956115 10.3389/fmicb.2021.746340 PMC8696170

[CR20] Kumar S, Stecher G, Li M, Knyaz C et al (2018) MEGA X: Molecular Evolutionary Genetics Analysis across computing platforms. Mol Biol Evol 2018;35:1547–1549. 10.1093/molbev/msy096 10.1093/molbev/msy096 PMC5967553 29722887

[CR22] Li W, Zhong Z, Song Y et al (2018) Human-pathogenic Enterocytozoon bieneusi in captive giant pandas (Ailuropoda melanoleuca) in China. Sci Rep 8:6590. 10.1038/s41598-018-25096-2 29700370 10.1038/s41598-018-25096-2 PMC5920105

[CR21] Li W, Xiao L (2020) Ecological and public health significance of Enterocytozoon bieneusi. One Health 12:100209. 10.1016/j.onehlt.2020.100209 33426263 10.1016/j.onehlt.2020.100209 PMC7779778

[CR23] López-Vélez R, Turrientes MC, Garrón C et al (1999) Microsporidiosis in travelers with diarrhoea from the tropics. J Travel Med 6:223–227. 10.1111/j.1708-8305.1999.tb00522.x 10.1111/j.1708-8305.1999.tb00522.x 10575169

[CR24] MatosO, Lobo ML, Xiao L (2012) Epidemiology of Enterocytozoon bieneusi infection in humans. J Parasitol Res 2012:981424. 10.1155/2012/981424 23091702 10.1155/2012/981424 PMC3469256

[CR25] Michlmayr D, Alves de Sousa L, Müller L et al (2020) Incubation period, spore shedding duration, and symptoms of Enterocytozoon bieneusi genotype C infection in a foodborne outbreak in Denmark, 2020. Clin Infect Dis 75:468–475. 10.1093/cid/ciab949 10.1093/cid/ciab949 PMC9427152 34791090

[CR26] Muadica AS, Messa AE Jr, Dashti A et al (2020) First identification of genotypes of Enterocytozoon bieneusi (Microsporidia) among symptomatic and asymptomatic children in Mozambique. PLoS Negl Trop Dis 14:e0008419. 10.1371/journal.pntd.0008419 32603325 10.1371/journal.pntd.0008419 PMC7357779

[CR27] Mutengo M, Dashti A, Liptáková M et al (2025) High prevalence of Enterocytozoon bieneusi (microsporidia) in asymptomatic schoolchildren, Zambia. Med Mycol 63:myaf065. 10.1093/mmy/myaf065 40693962 10.1093/mmy/myaf065

[CR28] Nei M, Kumar S (2000) Molecular Evolution and Phylogenetics. Oxford University Press, New York

[CR30] Öncü Öner T, Can H, Değirmenci Döşkaya A et al (2025) Molecular prevalence and genetic characterization of Enterocytozoon bieneusi in cattle in a dairy farm in Türkiye. BMC Vet Res 21:229. 10.1186/s12917-025-04701-3 40165244 10.1186/s12917-025-04701-3 PMC11956364

[CR29] Oğuz Kaya İ, Doğruman Al F, Mumcuoğlu İ (2018) Investigation of Microsporidia prevalence with calcofluor white and Uvitex 2B chemiluminescence staining methods and molecular analysis of species in diarrhoeal patients. Mikrobiyol Bul 52:401–412. Turkish. 10.5578/mb.67363 10.5578/mb.67363 30522425

[CR31] OnderZ, Yildirim A, Pekmezci D (2022) Occurrence and molecular characterization of Enterocytozoon bieneusi in water buffaloes (Bubalus bubalis) in Turkey. Acta Trop 233:106568. 10.1016/j.actatropica.2022.106568 35716763 10.1016/j.actatropica.2022.106568

[CR32] Öztürk EA, Al-Adilee YMS, Edwards W et al (2025) *Blastocystis* in humans, animals and the environment in a rural village of Türkiye, and crosstalk with the human intestinal microbiome. Front Microbiol 16:1665966. 10.3389/fmicb.2025.1665966 41190273 10.3389/fmicb.2025.1665966 PMC12580375

[CR33] Pekmezci D, Pekmezci GZ, Yildirim A et al (2019) Molecular detection of zoonotic microsporidia in domestic cats in Turkey: A preliminary study. Acta Parasitol 64(1):13–18. 10.2478/s11686-018-00003-x 30645737 10.2478/s11686-018-00003-x

[CR35] Pekmezci D, Yetismis G, Esin C et al (2020) Occurrence and molecular identification of zoonotic microsporidia in pet budgerigars (Melopsittacus undulatus) in Turkey. Med Mycol myaa088 10.1093/mmy/myaa088 10.1093/mmy/myaa088 33070189

[CR34] Pekmezci D, Yetismis G, Colak ZN et al (2021) First report and molecular prevalence of potential zoonotic Enterocytozoon bieneusi in Turkish tumbler pigeons (Columba livia domestica). Med Mycol 59:864–868. 10.1093/mmy/myab013 33724370 10.1093/mmy/myab013

[CR36] Sak B, Brady D, Pelikánová M et al (2011a) Unapparent microsporidial infection among immunocompetent humans in the Czech Republic. J Clin Microbiol 49:1064–1070. 10.1128/JCM.01147-10 21191056 10.1128/JCM.01147-10 PMC3067711

[CR37] SakB, Kváč M, Kučerová Z et al (2011b) Latent microsporidial infection in immunocompetent individuals - a longitudinal study. PLoS Negl Trop Dis 5:e1162. 10.1371/journal.pntd.0001162 21629721 10.1371/journal.pntd.0001162 PMC3101169

[CR38] Santín M, Fayer R (2009) Enterocytozoon bieneusi genotype nomenclature based on the internal transcribed spacer sequence: a consensus. J Eukaryot Microbiol 56:34–38. 10.1111/j.1550-7408.2008.00380.x 19335772 10.1111/j.1550-7408.2008.00380.x

[CR39] Şimşek NS, Cakmak I, Şimşek E (2025) First Data on the occurrence and genotyping of Enterocytozoon bieneusi in wrestling camels in Türkiye. Acta Parasitol 70:121. 10.1007/s11686-025-01061-8 40455338 10.1007/s11686-025-01061-8 PMC12129845

[CR40] Sürgeç E, Güvendi M, Karakavuk M et al (2023) Genotyping of Enterocytozoon bieneusi isolates detected in stray cats of İzmir, Türkiye. Parasitol Res 122:2729–2735. 10.1007/s00436-023-07974-5 37707609 10.1007/s00436-023-07974-5

[CR42] Taghipour A, Bahadory S, Javanmard E (2021) The global molecular epidemiology of microsporidia infection in sheep and goats with focus on Enterocytozoon bieneusi: a systematic review and meta-analysis. Trop Med Health 49:66. 10.1186/s41182-021-00355-7 34429166 10.1186/s41182-021-00355-7 PMC8385986

[CR41] Taghipour A, Bahadory S, Abdoli A (2022) A systematic review and meta-analysis on the global prevalence of cattle microsporidiosis with focus on Enterocytozoon bieneusi: An emerging zoonotic pathogen. Prev Vet Med 200:105581. 10.1016/j.prevetmed.2022.105581 35066319 10.1016/j.prevetmed.2022.105581

[CR43] Wang L, Xiao L, Duan L et al (2013) Concurrent infections of Giardia duodenalis, Enterocytozoon bieneusi, and Clostridium difficile in children during a cryptosporidiosis outbreak in a pediatric hospital in China. PLoS Negl Trop Dis 7:e2437. 10.1371/journal.pntd.0002437 24069491 10.1371/journal.pntd.0002437 PMC3772047

[CR44] Wang Y, Li XM, Yang X et al (2024) Global prevalence and risk factors of Enterocytozoon bieneusi infection in humans: a systematic review and meta-analysis. Parasite 31:9. 10.1051/parasite/2024007 38345479 10.1051/parasite/2024007 PMC10860563

[CR45] Yildirim A, Okur M, Uslug S et al (2020) First report on the molecular prevalence of Enterocytozoon bieneusi in horses in Turkey: genotype distributions and zoonotic potential. Parasitol Res 119:2821–2828. 10.1007/s00436-020-06783-4 32594238 10.1007/s00436-020-06783-4

