# Supplementary material for: One health survey of Enterocytozoon bieneusi in rural Adana (Türkiye) reveals zoonotic genotypes and two novel ITS genotypes in livestock
Source: Parasitol Res. 2026 Mar 28;125(1):53. doi: 10.1007/s00436-026-08662-w (PMC13056775; doi:10.1007/s00436-026-08662-w)
Supplement: Supplementary file 1 — Supplementary Material 1 [file 436_2026_8662_MOESM1_ESM.docx]

**Supplementary Table 1**. Distribution of *Enterocytozoon bieneusi* genotypes according to host species and sampled farms in the present study. ND indicates the samples with faint bands that were not confirmed as sequenced positives.

| Host | Genotype | Residence |
| --- | --- | --- |
| G1 | BEB6 | R27 |
| G19 | ShTrEb1 | R10 |
| C29 | J | R12 |
| C31 | Type IV | R23 |
| S1 | BEB6 | R27 |
| S2 | BEB6 | R27 |
| S13 | ShTrEb2 | R52 |
| S15 | ShTrEb2 | R34 |
| S29 | BEB6 | R6 |
| S35 | ShTrEb1 | R17 |
| S76 | BEB6 | R25 |
| S77 | BEB6 | R25 |

S32 ND R8

S39 ND R7

C50 ND R43

C59 ND R57

C65 ND R13

C72 ND R28

G38 ND R2

G42 ND R30
