# Supplementary material for: One health survey of Enterocytozoon bieneusi in rural Adana (Türkiye) reveals zoonotic genotypes and two novel ITS genotypes in livestock
Source: Parasitol Res. 2026 Mar 28;125(1):53. doi: 10.1007/s00436-026-08662-w (PMC13056775; doi:10.1007/s00436-026-08662-w)

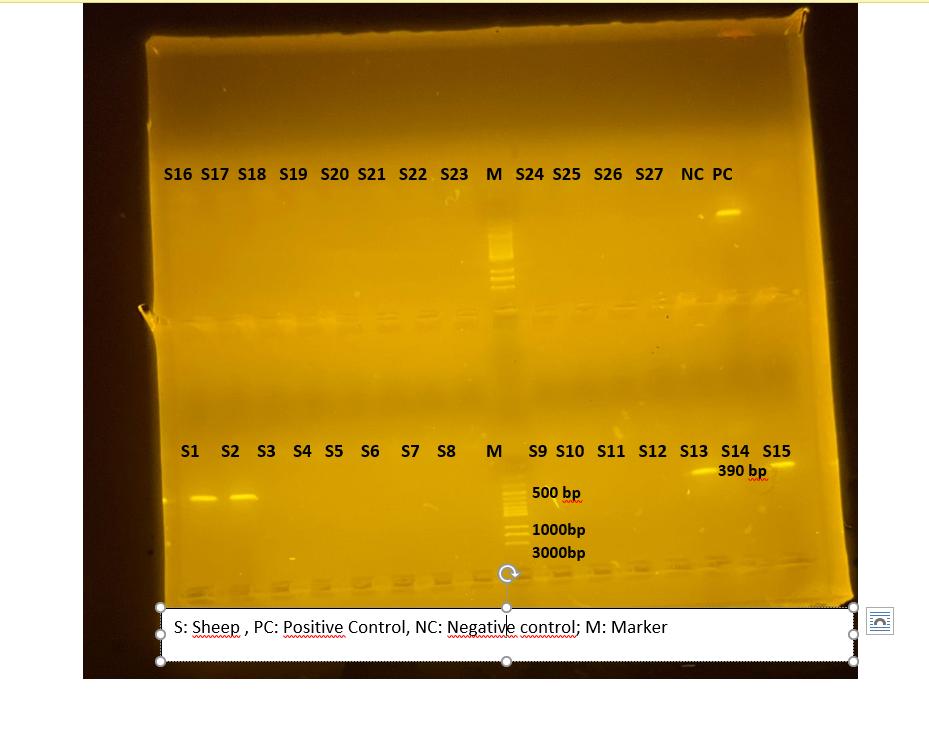


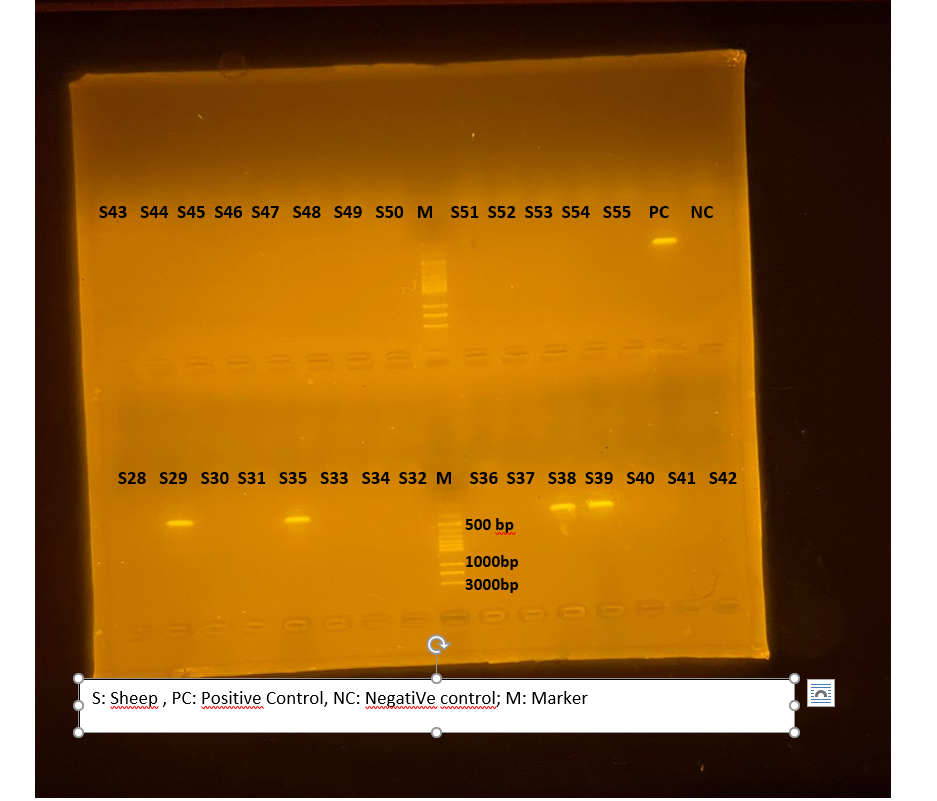


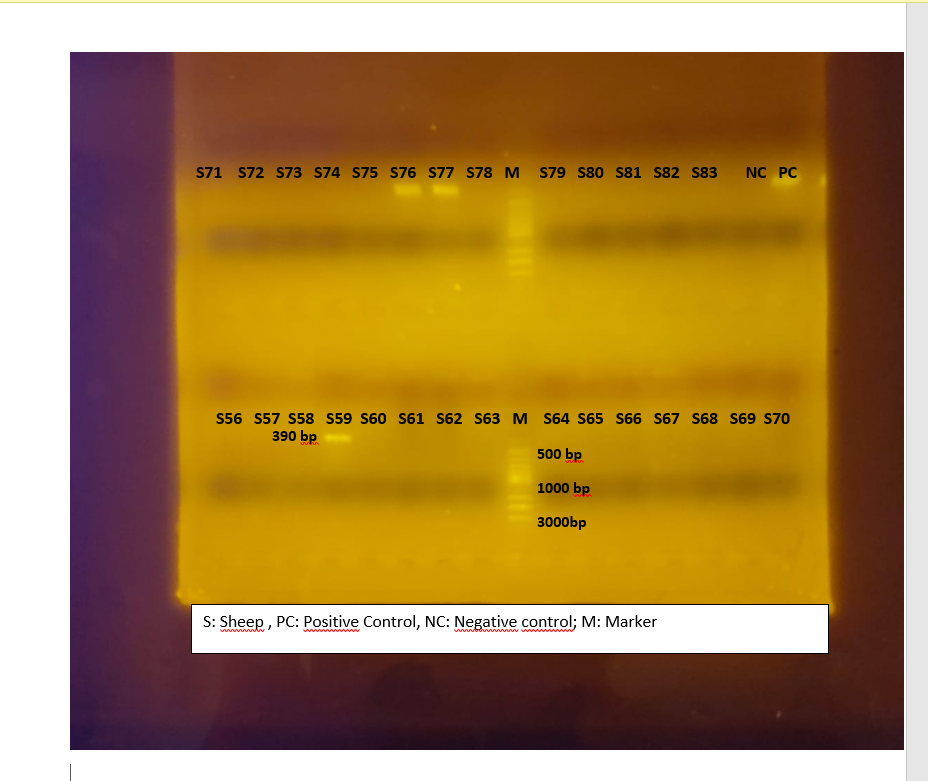


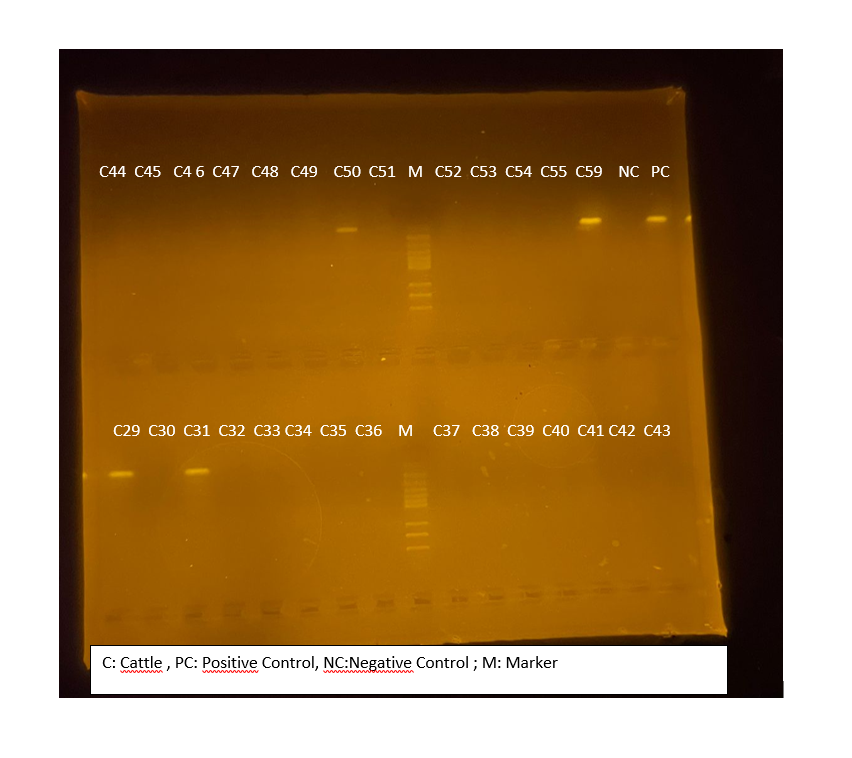


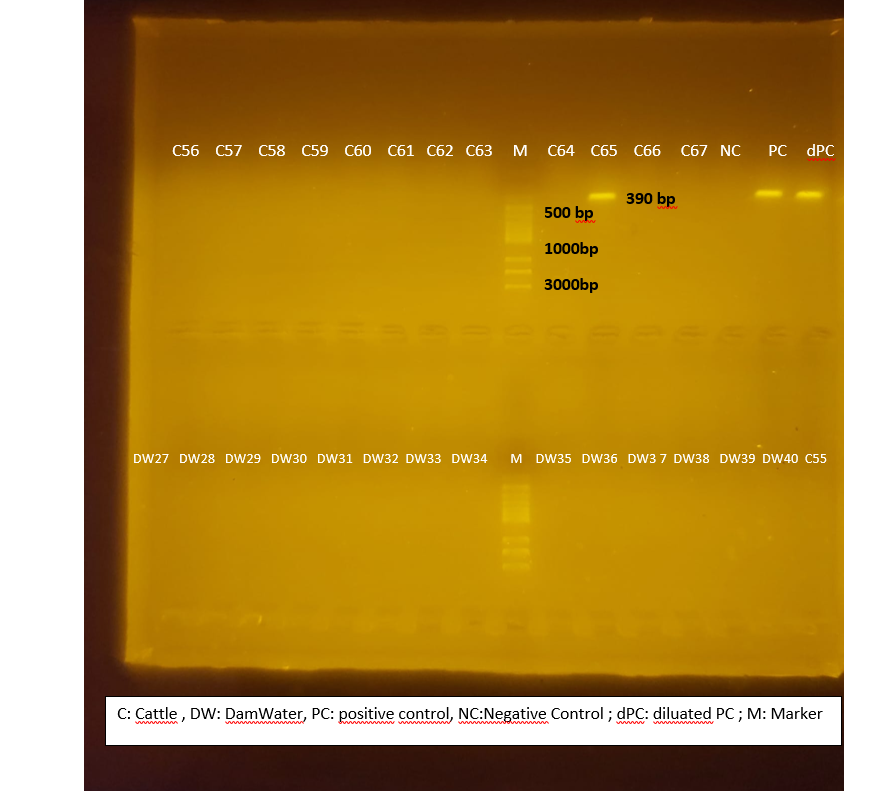


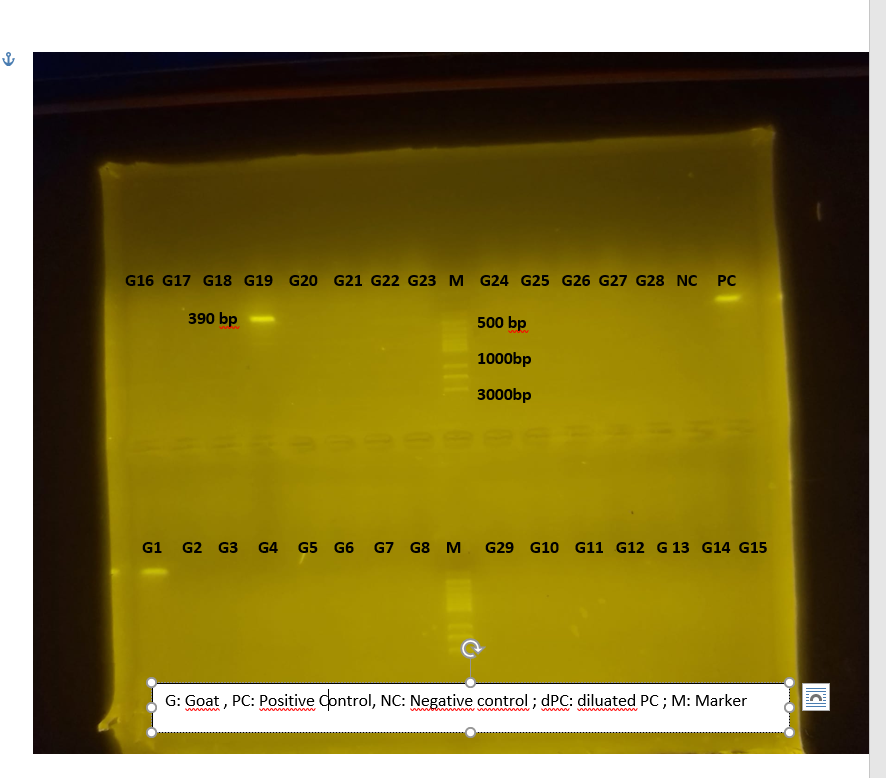


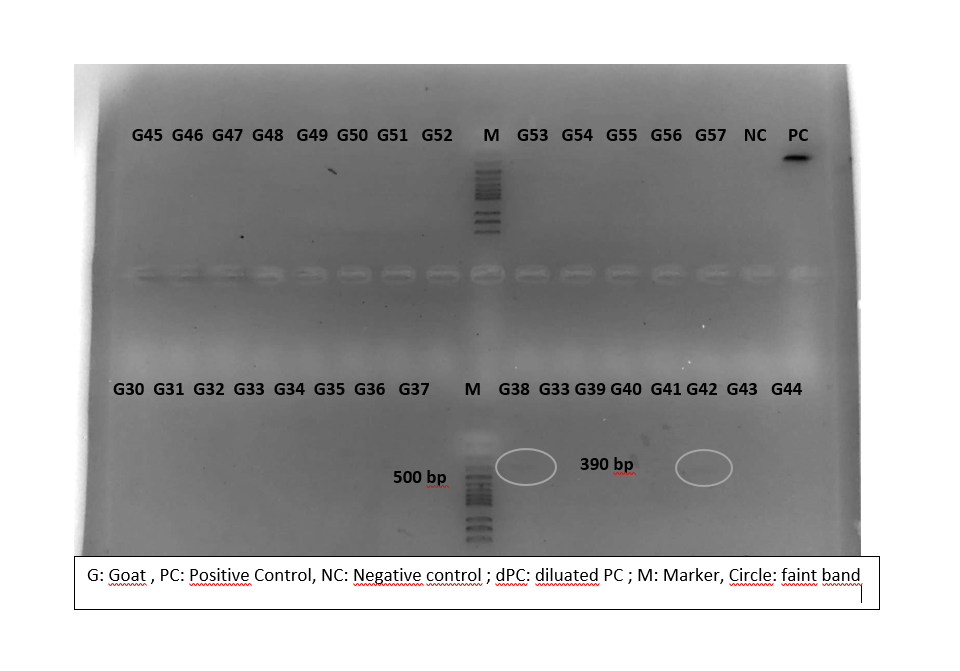


Human1-Human 28, This gel is representative of all samples analyzed, which were negative. NC: Negative Contorl, PC: Positive Contorl


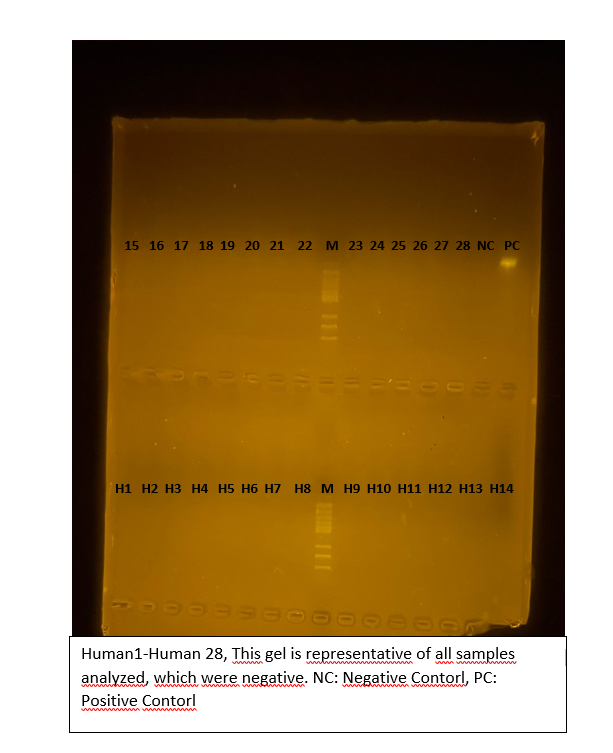

Supplement: Supplementary file 2 — Supplementary Material 2 [file 436_2026_8662_MOESM2_ESM.docx]
